# Editorial: Endogenous and exogenous factors influencing the function and metabolism of lipoproteins

**DOI:** 10.3389/fmolb.2022.1097963

**Published:** 2022-12-20

**Authors:** Carlo Cervellati, Domenico Sergi, Viviana Loria-Kohen, Alessandro Trentini

**Affiliations:** ^1^ Department of Translational Medicine and for Romagna, University of Ferrara, Ferrara, Italy; ^2^ VALORNUT Research Group, Department of Nutrition and Food Science, Faculty of Pharmacy, IdISSC, Complutense University of Madrid, Madrid, Spain; ^3^ Department of Environmental and Prevention Sciences, University of Ferrara, Ferrara, Italy

**Keywords:** Lp(a), systemic sclerosis, lipidomics, HDL, cardiovascular diseases

Alterations in the levels and composition of lipoproteins are associated with several diseases (Fernandes Das Neves et al., 2021). The classical view that these complex particles are mere carriers of lipids and are solely implicated in atherosclerosis is now considered amply outdated. For instance, the levels of routinely assessed plasma total cholesterol, low density lipoprotein-cholesterol (LDL-C), high-density lipoprotein-cholesterol (HDL-C), and triglycerides are now regarded as strong risk factors for a plethora of diseases including, but not limited to, neurodegenerative diseases and auto-immune disorders (Marsillach et al., 2020).

Alterations in the circulating lipid profile, termed dyslipidemia, is a well-known risk factor for cardiovascular diseases (CVDs). In this context, the lipoproteins classically associated with CVDs have been recently complemented with Lp(a). Within this framework, (Cremonini et al.) evaluated the ability of Lp(a) to identify subjects with more severe carotid atherosclerosis (CA) in a population of individuals with metabolic syndrome (MetS). They found that higher levels of Lp(a) were independently associated with severe CA when measured by carotid intima media thickness (cIMT) or the number of atherosclerotic plaques. However, these results need further validation in a larger cohort.

Non-etheless, this may be a starting point for future studies aiming at the implementation of this marker for CV risk stratification even in both primary and secondary prevention of CVDs in subjects affected by MetS. Indeed, despite Lp(a) being increasingly acknowledged as a “non-classical” cardiovascular risk factor (Reyes-Soffer et al., 2022), its levels are not routinely assessed in the clinic nor used to stratify risk due to lack of consensus in analytical methods.

Not only patients affected by CVDs or by MetS suffers from dyslipidemia, but also those with systemic sclerosis (SSc), which has been the focus of a review by (Gogulska et al.). The authors reported differences in the lipid profile of individuals affected by SSc which are marked by an increase in Lp(a) and triglycerides relative to healthy individuals. On the contrary, the impact of SSc on total, LDL and HDL cholesterol remains a matter of contention with both an increase as well as a decrease in these lipoproteins being reported in individuals affected by SSc. Along with the aberrant circulating lipid profile, SSc individuals also present defective lipid metabolism at the cellular level as indicated by alterations in the physiological concentrations of acyl-glycine, fatty acids and carnitine derivatives (Gogulska et al.). The aberrations in lipid metabolism which characterizes SSc also impact cell plasma membranes of SSc patients, which are characterized by an increase in the cholesterol:phospholipid ratio and by a drop in the unsaturation index (Gogulska et al.). In light of the above considerations on lipid metabolism, authors advocate the use of lipidomics as a powerful tool to identify and unlock novel therapeutic strategies to tackle SSc. Indeed, the lipid composition of lipoprotein subclasses is a key discriminant in dictating their function and apolipoprotein content. As such, the relative content of the different phospholipids, such as phosphatidylcholines, sphingomyelins and phosphatidylethanolamines influence the binding of apolipoproteins to the particle. In turn, the presence of different combinations of these proteins modulates the functional properties of the lipoprotein subclasses. These considerations particularly apply to HDL, which is the most heterogenous family of circulating lipoproteins (Rosenson et al., 2016). One of the most studied functions of HDL particles is their ability to stimulate cholesterol efflux from lipid laden cells (named cholesterol efflux capacity, CEC) participating in reverse cholesterol transport (RCT). Thus, factors that can positively or negatively modulate this activity could have a tremendous impact on cardiovascular risk regardless of HDL-c concentration. In a work included in this issue, (Palumbo et al.) explored the effect of PCSK9 inhibitors, known LDL lowering drugs, on HDL CEC. After 6 months of treatment, the authors compared the ability of HDL to stimulate the cholesterol efflux through ABCG1, ABCA1 and by aqueous diffusion with values obtained before the treatment. What they found is that the treatment with PCSK9 inhibitors *in vivo* was able to increase ABCG1 and aqueous diffusion mediated cholesterol efflux, despite the lack of changes in HDL cholesterol levels. This adds to the previously documented pleiotropic effects of PCSK9 inhibitors (Basiak et al., 2021), suggesting that their positive contribution to cardiovascular homeostasis may also go through the modulation of HDL functionality.

As extensively discussed, circulating lipids and their metabolism are key variables impacting human health, particularly with regard to cardiometabolic health. Moreover, it must be acknowledged that other parameters known to shape cardiometabolic health, like body weight and adiposity, also significantly affect the circulating lipid profile. In this regard, *Zhu et al.* investigated the changes in the circulating lipid profile and underlying influencing factors in the population of a city in central China (Zhu et al.). In this study, authors reported that age was a more important discriminant in shaping the circulating lipid profile in females, which instead was more profoundly influenced by BMI in males. However, as expected, independently from age and gender, the presence of diabetes should always be regarded as a pivotal driver of dyslipidemia (Zhu et al.).

From what is reported in this Research Topic, it is clear that both exogenous (e.g. pharmacological treatments, diet, life-style habits) and endogenous factors (e.g. genetic susceptibility, epigenetics, disease state) can modulate the functionality and metabolism of all lipoproteins. Collectively, such factors may contribute to the onset and progression of deleterious chronic states, such as oxidative stress and inflammation. These conditions represent the main culprit driving the change in function and biological role of HDL, and of the formation of oxidized LDL (Poznyak et al., 2020). Thus, the study of modifiable factors that could impact lipoprotein functionality and metabolism, may help in identifying possible intervention strategies to overcome the risk of developing diseases.
